# Growth of *in vitro Oncidesa* plantlets cultured under cold cathode fluorescent lamps with super-elevated CO_2_ enrichment

**DOI:** 10.1093/aobpla/plt044

**Published:** 2013-09-27

**Authors:** Atsushi Norikane, Jaime A. Teixeira da Silva, Michio Tanaka

**Affiliations:** 1 Department of Applied Biological Science, Faculty of Agriculture, Kagawa University, Miki-cho, Kagawa 761-0795, Japan; 2 Present address: PO Box 7, Miki-cho Post Office, Ikenobe 3011-2, Kagawa-ken 761-0799, Japan

**Keywords:** CCFL, *Oncidesa* (formerly *Oncidesa* Gower Ramsey ‘U-1’), photoautotrophic growth, single-leaf photosynthesis, super-elevated CO_2_

## Abstract

As interest in how to increase biomass production through biotechnological means gains traction, focus is turning towards the use of photoautotrophic micropropagation under elevated levels of carbon dioxide (CO_2_) to maximize plant growth and productivity. The effect of super-elevated CO_2_ with cold cathode fluorescent lamps (CCFLs) on the photoautotrophic growth of *Oncidesa in vitro* has been studied using a gas-permeable film culture vessel, the ‘Vitron’. The growth of *Oncidesa* (formerly *Oncidesa* Gower Ramsey ‘U-1’) plantlets on Vacin and Went (VW) medium was stimulated by 10 000 μmol mol^−1^ CO_2_. In particular, increasing the photosynthetic photon flux density (PPFD) from 45 to 60 μmol m^−2^ s^−1^ under 10 000 μmol mol^−1^ CO_2_ in the growth chamber remarkably increased the number of leaves and roots, and shoot and root fresh and dry weights compared with plantlets under the same level of CO_2_ under low PPFD (45 μmol m^−2^ s^−1^). However, there was a remarkable decrease in photosynthetic capacity, and chlorosis and browning of leaves. In stark contrast, plantlets grown on Kyoto medium at 10 000 μmol mol^−1^ CO_2_ under high PPFD had a higher photosynthetic rate than plantlets grown on VW medium, and no chlorosis or browning was observed. Furthermore, shoot growth was remarkably enhanced. Therefore, super-elevated CO_2_ (10 000 μmol mol^−1^) enrichment and growth under CCFLs can positively affect the efficiency and quality of commercial production of clonal *Oncidesa* plantlets.

## Abstract (precis)

How can one increase the biomass of plants, particularly orchids, in an effective way? Photoautotrophic micropropagation is an effective means of increasing the biomass of *Oncidesa* orchids. Using 10,000 µmol mol^−1^ CO_2_ and a photosynthetic photon flux density of 60 µmol m^−2^ s^−1^, the number of leaves and roots and shoot and root fresh and dry weights could be considerably increased, but only when cultured on Kyoto medium rather than Vacin and Went medium. Super-elevated CO_2_ enrichment and growth under cold cathode fluorescent lamps can positively affect the efficiency and quality of commercial production of clonal *Oncidesa* plantlets.

## Introduction

The use of plant tissue culture as a way to increase plant biomass in a short span of time is an attractive application of biotechnology practised by many plant scientists. The ability to induce greater biomass by increasing the carbon dioxide (CO_2_) concentration through photoautotrophic micropropagation—which has proven benefits in terms of productivity (Kozai *et al.* 2005)—has wide practical applications for ornamental and other horticultural crops.

*Oncidium* is an epiphytic and terrestrial orchid. This genus comprises about 400 species distributed mainly in tropical and subtropical South and Central America (Endress 1996). Many hybrid *Oncidium* have been produced, the most attractive having become commercially important as potted plants and cut flowers. Fast (1973) first reported that *Oncidium* plantlets could be induced by shoot tips, and several effective protocols for clonal propagation have been developed since then (Arditti 2008) and are now used for commercial micropropagation. However, relatively high production costs continue to hamper the expansion of hybrid *Oncidium* production since it may take almost one year for plantlets to reach the acclimatization stage. Since orchids such as *Oncidium* are inherently slow growers, there are considerable energy costs spent on controlling air temperature and lighting in the culture room. To overcome these limitations, despite several studies existing on efficient multiplication and regeneration of *Oncidium* plantlets (e.g. Chen and Chang 2000; Chen *et al.* 2001; Jheng *et al.* 2006), it is also important to enhance the growth of plantlets regenerated *in vitro*.

Over two decades, many studies have been conducted on photoautotrophic culture in various species, including *Oncidium* hybrids (He *et al.* 2003), which has several advantages over photomixotrophic culture: promotion of growth and photosynthesis, high survival percentage *ex vitro*, elimination of physiological and morphological disorders, and less microbial contamination (reviewed in Xiao *et al.* 2011). Under photoautotrophic culture, CO_2_ is one of the most important factors directly affecting the growth and photosynthesis of plantlets because they should produce complex organic compounds from CO_2_ as a carbon source using energy from light. Therefore, it is necessary for enhancing photoautotrophic growth to provide a sufficient optimal concentration of CO_2_. Tanaka (1991) developed several film culture vessels that had high gas and light permeability. The photoautotrophic growth of several orchid plantlets was stimulated using 3000 μmol mol^−1^ CO_2_ enrichment: C_3_ *Cymbidium* (Tanaka *et al.* 1999; Teixeira da Silva *et al.* 2007), C_3_ *Epidendrum* (Giang and Tanaka 2004), CAM *Phalaenopsis* (Tanaka *et al.* 1993) and C_3_ *Oncidium* (He *et al.* 2003). It was previously demonstrated in CAM *Phalaenopsis* that an increase in CO_2_ concentration from 3000 to 5000 μmol mol^−1^ remarkably enhanced the photoautotrophic growth of plantlets in film culture vessels (Norikane and Tanaka 2010), implying that higher CO_2_ enrichment (>3000 μmol mol^−1^) could have a positive effect on the photoautotrophic growth of orchid plantlets.

Hew *et al.* (1995) and Gouk *et al.* (1997) conducted detailed plant physiological studies on the effect of super-elevated CO_2_ levels (10 000 μmol mol^−1^) on the *in vitro* growth of CAM orchids Mokara White and Mokara Yellow, concluding that these levels were able to stimulate the growth of plantlets on sugar-added medium (i.e. photomixotrophic conditions). A similar trend was noticed for the *Cymbidium* hybrid but at a lower CO_2_ level (3000 μmol mol^−1^) (Teixeira da Silva *et al.* 2007). In the photoautotrophic culture of *Cymbidium*, 10 000 μmol mol^−1^ CO_2_ enrichment remarkably enhanced the growth of plantlets in a conventional glass bottle with a TPX cap having a hole covered with a gas-permeable membrane (Norikane *et al.* 2010). However, beyond these studies, little is known about the effect of super-elevated CO_2_ enrichment on the photoautotrophic growth of orchid plantlets (or any other crop for that matter) in film culture vessels. Thus, one key objective of our study was to ascertain whether super-elevated CO_2_ would have further positive effects on the photoautotrophic growth of *Oncidesa* plantlets in film culture vessels.

Light is also an essential factor for plant growth and development. Recently, the use of cold cathode fluorescent lamps (CCFLs) as a radiation source for plants has been attempted because of their attractive features, including their small diameter (1.6–3.0 mm), long life (50 000 h) and low heat generation, with an estimated 40 % reduction in electric energy consumption used for lighting and air conditioners during micropropagation (Tanaka *et al.* 2009; Norikane and Tanaka 2010). Those studies demonstrated that *Cymbidium* and *Phalaenopsis in vitro* plantlets cultured under CCFLs showed enhanced photoautotrophic growth compared with plantlets cultured under conventional heat fluorescent lamps. Ding *et al.* (2010) and Wang *et al.* (2011) also reported the usefulness of CCFLs as a lighting source for the micropropagation of tree peony and *Gerbera*. Our present study is the first report on the use of CCFLs as a radiation source for *Oncidesa* plantlet growth *in vitro*.

The aim of the present study was to achieve more efficient and higher-quality commercial clonal orchid plantlets, in this case, an *Oncidium* hybrid (*Oncidesa*), by super-elevated CO_2_ enrichment under CCFLs on two different media.

## Methods

### Plant materials

The explants used in this study were shoots with 2–3 leaves obtained from a mass of protocorm-like bodies of *Oncidesa* (formerly *Oncidesa* Gower Ramsey ‘U-1’; Royal Horticultural Society (RHS) 2013) derived from shoot-tip culture. This is a sympodial orchid hybrid, thin-leaved and with a C_3_ mode of photosynthesis (Hew and Yong 1997). Twenty-five shoots were cultured in each culture vessel for 3 months, and two culture vessels were used for each treatment.

### Culture medium

Vacin and Went (VW) (Vacin and Went 1949) sugar-free liquid medium was used as the basal medium. To examine the effect of basal medium under super-elevated CO_2_ enrichment, Kyoto (Tsukamoto *et al.* 1963) sugar-free liquid medium was also used. VW and Kyoto media are two of the most commonly used media in orchid biotechnology (Teixeira da Silva *et al.* 2005). The pH of the media was adjusted to 5.3 with 1 N NaOH or HCl before autoclaving at 121 °C for 17 min.

### Preparation of the ‘Vitron’ rockwool system

The film culture vessel ‘Vitron’ (122 mm × 122 mm × 140 mm) consists of a three-dimensional injection-moulded polypropylene frame covered by a heat-sealed OTP film (Otsuka Techno Co. Ltd, Tokushima, Japan) on all sides, except the top (Giang and Tanaka 2004). OTP is a multi-layer film consisting of three layers: the outer layer of TPX (4-methyl-1-pentane polymer) and the inner layer of CPP (a polypropylene) which are bonded together by a middle layer of polyolefin resins. The top seal film (OTP) is affixed to the top of the vessel after removing the paper backing to expose the adhesive. The medium substrate was rockwool (a 25 joined block, 5 × 5, of Grodan^®^ Rockwool Multiblock™ AO 18/30, Grodania A/S, Denmark) with 180 mL of liquid medium. The rockwool was previously sterilized in a dry sterilizer (150 °C, 1 h), and placed in the ‘Vitron’. Then, sterilized liquid medium was poured evenly over the rockwool.

### Culture conditions

**Figure 1.**
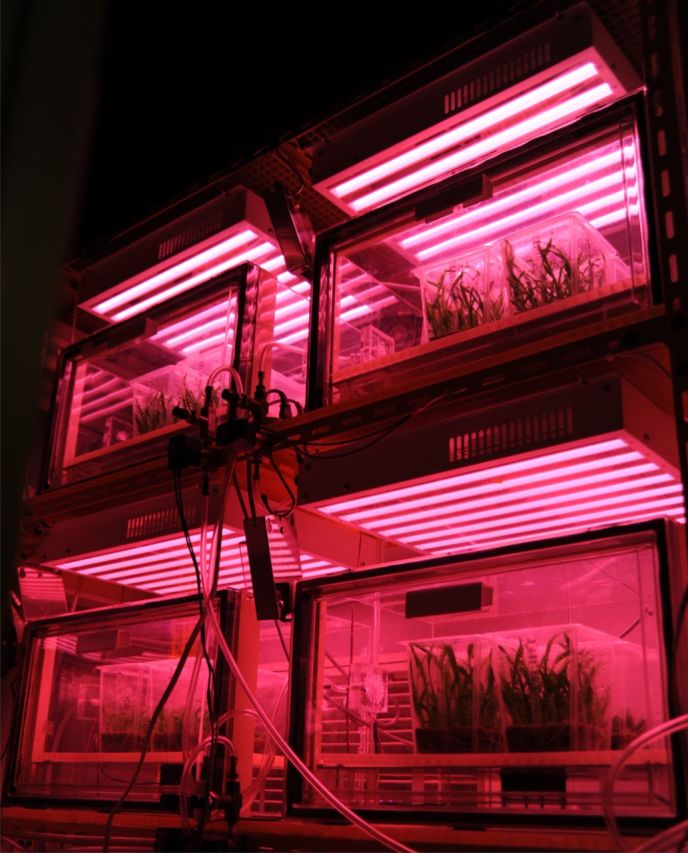
CO_2_ enrichment system under a CCFL light source.

The culture conditions were 25 ± 1 °C, a 16-h photoperiod, and a photosynthetic photon flux density (PPFD) of 45 and 60 μmol m^−2^ s^−1^ (R/B ratio: 80 % red (∼660 nm) + 20 % blue (∼450 nm), a conventional CCFL light unit; NK System, Osaka, Japan). CO_2_ enrichment was 380 (ambient/control), 3000, 5000 or 10 000 μmol mol^−1^. Experiments were conducted under each CO_2_ concentration by placing the vessels in different transparent acrylic desiccation chambers (Fig. 1) in which the CO_2_ concentration inside the chamber was controlled with an infrared CO_2_ controller (ZEP 9, Fuji Electric Co., Ltd, Japan) and a CO_2_ gas inlet line (Tanaka *et al.* 1992). CO_2_ was injected into the chamber from a pure source through a solenoid valve and a microneedle valve. To prevent air stratification inside the chambers, a tube axial DC fan was fitted to the centre of a false floor and a conventional CCFL light source was installed on the roof of the chamber (Fig. 1).

### Measurement of growth parameters

The number of leaves and roots, plant height, pseudobulb volume, root length, shoot, pseudobulb and root fresh weights, shoot, pseudobulb and root dry weights, pseudobulb formation frequency and the soil plant analysis development (SPAD) value of leaves of plantlets grown *in vitro* were recorded after 90 days. The pseudobulb formation frequency was calculated as a percentage of the plantlets that formed a pseudobulb. Pseudobulb volume (*V*_p_) was calculated as an ellipsoid:
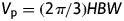

where *H*, *B* and *W* are pseudobulb height, breadth and width, respectively (Winkler *et al.* 2009). The SPAD value of leaves was measured with a chlorophyll meter (SPAD-502, Minolta Co., Ltd, Osaka, Japan) in the second leaf, counted from the top downward, of plantlets.

### Measurement of photosynthesis

The photosynthetic light response curve and net photosynthetic rate were measured in the second leaf, counting from the top downwards, of plantlets, in which a pseudobulb was not formed, after culturing for 90 days. It was measured in at least five plants using a portable infrared gas analyser (LI-6400, Li-COR, Lincoln, NE, USA). For obtaining the photosynthetic light response curve, the photon flux density that was provided from a red LED light source built into the top of the leaf chamber was changed from 300 to 0 μmol m^−2^ s^−1^. The net photosynthetic rate was measured at 300 μmol m^−2^ s^−1^ (saturating or near-saturating PPFD). The CO_2_ concentration and temperature in the leaf chamber were adjusted to maintain 400 μmol mol^−1^ and 25 °C, respectively. The relative humidity in the leaf chamber was kept as close to 65–70 % as possible. The air flow rate was 200 mL min^−1^.

### Statistical analysis

Means were separated by ANOVA and significant differences were assessed by Tukey's multiple range test and a Student's *t*-test at *P* = 0.05.

## Results and Discussion

Elevated CO_2_ increases the dry mass of plants (Mortensen 1987). Assimilate partitioning to the roots under elevated CO_2_ has also been shown for a wide range of herbaceous species (Farrar and Williams 1991). Regarding the micropropagation of orchids, high CO_2_ enrichment in *Cymbidium* (Tanaka *et al.* 1999; Teixeira da Silva *et al.* 2007) and *Phalaenopsis* (Norikane and Tanaka 2010) or super-elevated CO_2_ enrichment in Mokara Yellow (Hew *et al.* 1995) and *Cymbidium* (Norikane *et al.* 2010) increased the dry weight, especially in roots, playing a role as a sink. In our present study, the enhanced root growth of plantlets in the ‘Vitron’ was also observed with an increase in CO_2_ concentration from 380 (non-CO_2_ enriched) to 10 000 μmol mol^−1^ under both low and high PPFD; the maximum number of roots, root length, and root fresh and dry weights were obtained when plantlets were grown under 10 000 μmol mol^−1^, regardless of PPFD level (Table 1). The enhanced root growth of *in vitro* plantlets as a result of super-elevated CO_2_ enrichment might enhance *ex vitro* growth through the acquisition of essential resources that would increase the carbohydrate sink that would accumulate in the root and be utilized when these plantlets are transferred to the greenhouse. On the other hand, our study showed that an increase in CO_2_ concentration remarkably increased plantlet root and shoot weights; maximum shoot fresh and dry weights were obtained when plantlets were grown under 10 000 μmol mol^−1^ CO_2_ with low or high PPFD, respectively (Table 1). Plantlets grown at 10 000 μmol mol^−1^ CO_2_ under both levels of PPFD also had larger and heavier *in vitro*-formed pseudobulbs than those at other CO_2_-enrichment conditions, although the frequency of formation did not differ greatly (Table 1). It therefore seems that the plantlets had the heaviest shoot weight as a result of the accumulation of carbohydrates in the pseudobulb as a direct consequence of photosynthesis. Young plants of sympodial thin-leaved *Oncidium* usually produce a new shoot from an axillary bud at the second node under the pseudobulb (Tanaka *et al.* 1986), so, for the shoot to develop, the pseudobulb acts as a source of photosynthate (Hew and Ng 1996). Therefore, increasing pseudobulb weight and volume by super-elevated CO_2_ enrichment influences the formation and rapid development of the new shoot.

**Table 1.** Effects of CO_2_ concentration and PPFD on the *in vitro* growth of *Oncidesa* plantlets under CCFL. ^x^Chlorophyll content in the second leaf, counted from the top downwards, of the plantlets. ^y^Different letters within a column indicate significant differences at *P* ≤ 0.05 by Tukey's multiple range test. *n* = 50. ^z^Non-CO_2_ enrichment.

PPFD (μmol m^−2^ s^−1^)	CO_2_ concentration (μmol mol^−1^)	No. of leaves	No. of roots	Plant height (cm)	Pseudobulb volume (cm^3^)	Root length (cm)	Fresh weight (mg)			Dry weight (mg)			Pseudobulb formation frequency (%)	Chlorophyll content^x^ (SPAD value)
							Shoot	Pseudobulb	Root	Shoot	Pseudobulb	Root		
45	Ambient^z^	6.3d^y^	0.9e	5.8c	0.2b	0.4d	293.1g	36.0d	8.1e	16.8f	1.0b	0.3e	11.1	25.0c
	3000	7.1bc	2.4d	8.6b	0.8b	3.9c	473.0ef	189.7cd	108.7d	29.5e	7.3b	5.0d	31.8	32.3ab
	5000	7.7b	3.3c	11.7a	1.5ab	5.1b	600.6d	355.3bc	180.7c	38.8cd	12.1b	8.7c	14.6	30.8b
	10 000	7.4b	4.1b	11.0ab	2.4a	7.4a	957.6b	566.3a	324.0b	62.9b	21.6a	16.9b	47.3	33.5a
60	Ambient	6.4cd	1.2e	5.7c	–	0.3d	387.1fg	–	11.8e	24.1ef	–	0.5e	0	26.8c
	3000	7.4b	2.4d	9.6ab	0.7b	3.6c	574.3de	183.7cd	96.1d	37.3d	6.8b	4.8d	38.9	31.8ab
	5000	7.4b	3.1c	10.6ab	1.2b	4.8b	703.3c	304.6cd	161.7c	45.5c	10.8b	7.8c	29.5	32.6ab
	10 000	8.5a	5.8a	11.1a	2.4a	7.4a	1131.6a	560.6ab	410.0a	93.1a	28.0a	25.3a	32.6	30.9b

In *Cymbidium*, an increase in PPFD (using CCFLs) under 10 000 μmol mol^−1^ CO_2_ enrichment further enhanced the *in vitro* growth of both shoots and roots (Norikane *et al.* 2010). A similar trend was observed in *Oncidesa* plantlets in the present study; the number of leaves and roots, and shoot and root fresh and dry weights of the plantlets grown at 10 000 μmol mol^−1^ CO_2_ under high PPFD increased remarkably, although plant height, root length, and pseudobulb volume, fresh and dry weights and formation frequency did not differ between both PPFD levels under 10 000 μmol mol^−1^ CO_2_ (Table 1). Thus, we concluded that super-elevated CO_2_ enrichment (10 000 μmol mol^−1^ in the ‘Vitron’) with high PPFD (provided by CCFLs) also has a positive effect on the growth of both shoots and roots in *Oncidesa*.

**Figure 2.**
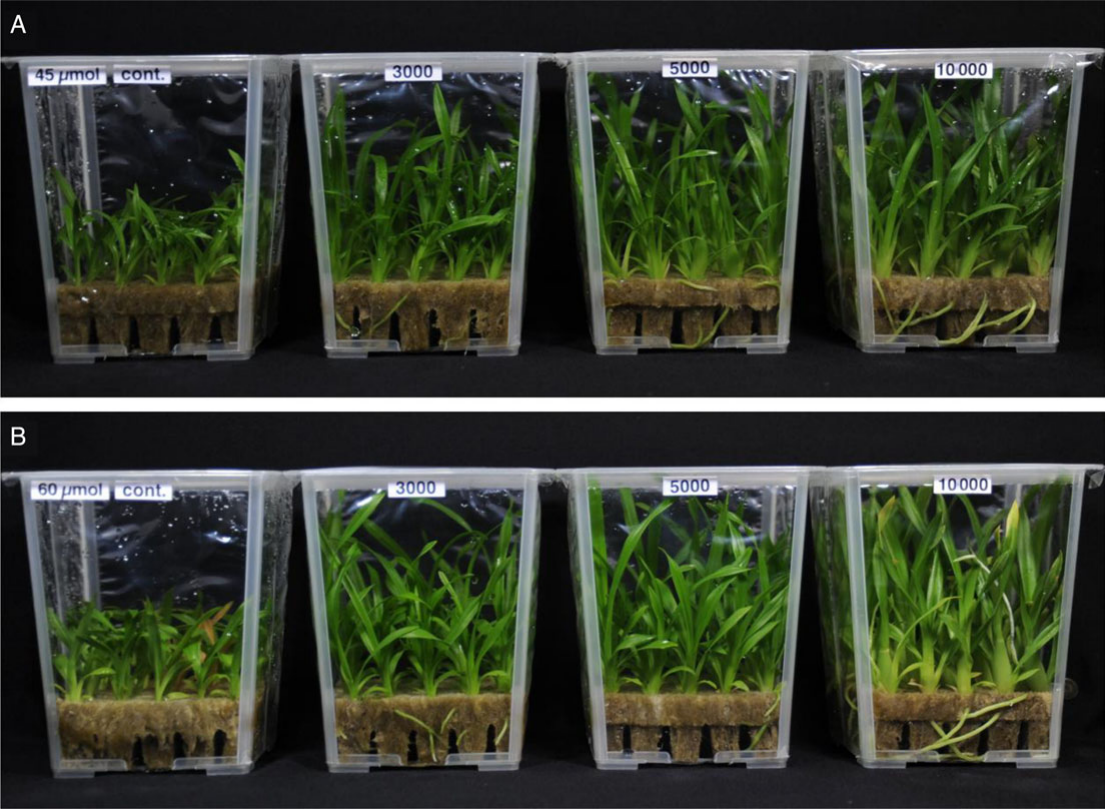
Effects of CO_2_ concentration and PPFD on the *in vitro* growth of *Oncidesa* plantlets under CCFLs. (A) Low PPFD (from left to right: ambient, 3000, 5000 and 10 000 μmol mol^−1^). (B) High PPFD (from left to right: ambient, 3000, 5000 and 10 000 μmol mol^−1^). (A) and (B) are at the same scale.

High CO_2_ and super-elevated CO_2_ enrichment tend to cause foliar symptoms such as chlorosis, necrosis or bleaching in several plant species (Mortensen 1987; Wheeler *et al.* 1993; Mackowiak and Wheeler 1996; Sicher 2008; Croonenborghs *et al.* 2009). Leaf yellowing is attributed to photoinhibition, nutrient deficiency, premature senescence and other causes (Cook *et al.* 1998; Sicher 1998, 2008). Norikane *et al.* (2010) also indicated that chlorosis could be observed in *Cymbidium* plantlets grown at 10 000 μmol mol^−1^ CO_2_ under high PPFD in all leaf tips except for new leaves; furthermore, the leaf tips of these plantlets withered and died after transferring them to the greenhouse for acclimatization and growth *ex vitro*. In the present study, *Oncidesa* plantlets at 10 000 μmol mol^−1^ CO_2_ under high PPFD showed remarkably enhanced growth and reduced chlorophyll content (SPAD value) compared with plantlets at the same CO_2_ concentration under low PPFD (Table 1), and severe chlorosis in the whole leaf blade and browning in part of the leaf blade were observed from a late stage of culture, although no such symptoms were observed in plantlets grown at 10 000 μmol mol^−1^ CO_2_ under low PPFD, nor at 3000 and 5000 μmol mol^−1^ CO_2_ under both PPFDs (Fig. 2). This may negatively affect the *ex vitro* growth of plantlets that were cultured at 10 000 μmol mol^−1^ CO_2_ under high PPFD.

**Figure 3.**
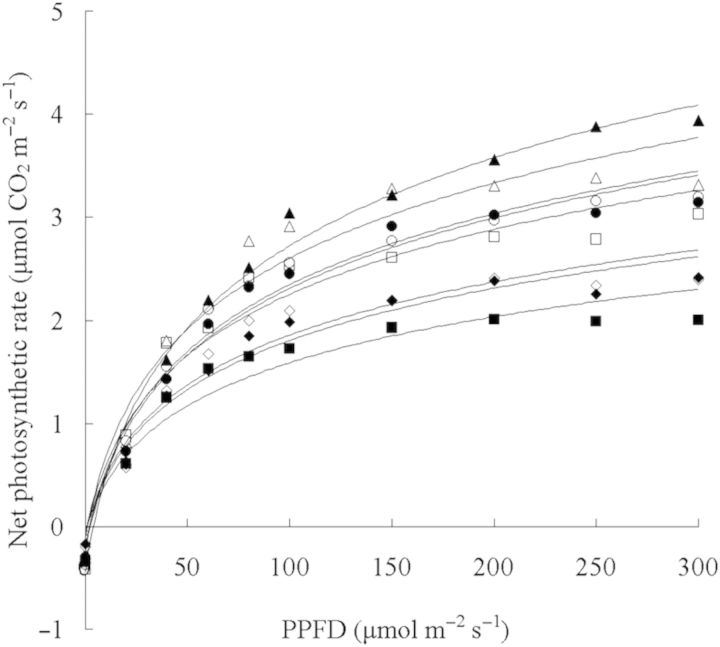
Photosynthetic light–response curve for single *Oncidesa* plantlet leaves grown at 0, 3000, 5000 and 10 000 μmol mol^−1^ CO_2_ enrichment under low and high PPFD. Open diamonds: non-CO_2_ enrichment under low PPFD; closed diamonds: non-CO_2_ enrichment under high PPFD; open circles: 3000 μmol mol^−1^ CO_2_ enrichment under low PPFD; closed circles: 3000 μmol mol^−1^ CO_2_ enrichment under high PPFD; open triangles: 5000 μmol mol^−1^ CO_2_ enrichment under low PPFD; closed triangles: 5000 μmol mol^−1^ CO_2_ enrichment under high PPFD; open squares: 10 000 μmol mol^−1^ CO_2_ enrichment under low PPFD; closed squares: 10 000 μmol mol^−1^ CO_2_ enrichment under high PPFD.

C_3_ plants growing in long-term elevated CO_2_ showed a decline in photosynthetic capacity (Gunderson and Wullschleger 1994; Sage 1994; Drake *et al.* 1997), which may reduce plant growth. Our previous study on super-elevated CO_2_ enrichment *in vitro* indicated that *Cymbidium* plantlets grown at 10 000 μmol mol^−1^ CO_2_ under high PPFD showed decreased photosynthetic capacity and total Rubisco activity tended to decline, possible factors explaining the decreasing photosynthetic capacity (Norikane *et al.* 2010). In the present study the net photosynthetic rate of single leaves of *Oncidesa* was measured at saturating or near-saturating PPFD (200–300 μmol m^−2^ s^−1^) at the end of the culture period. At low PPFD, even in plantlets grown at 10 000 μmol mol^−1^ CO_2_, a decrease in the net photosynthetic rate of single leaves did not occur, while at high PPFD, plantlets grown at the same high level of CO_2_ showed a significant decrease (Fig. 3). The latter value was similar to that of plantlets grown at non-CO_2_ enrichment under high PPFD, in which almost no growth and browning of leaves were observed. This reduction may be due to damage of the photosynthetic apparatus rather than the photosynthetic acclimation response to elevated CO_2_ (Sage 1994) because browning was observed in plantlets' leaves (Fig. 2). This might also negatively impact the *ex vitro* growth of plantlets cultured at 10 000 μmol mol^−1^ CO_2_ under high PPFD. Therefore, super-elevated CO_2_ enrichment as a method to improve the culture of *Oncidesa in vitro* must be further refined for it to be effective.

**Figure 4.**
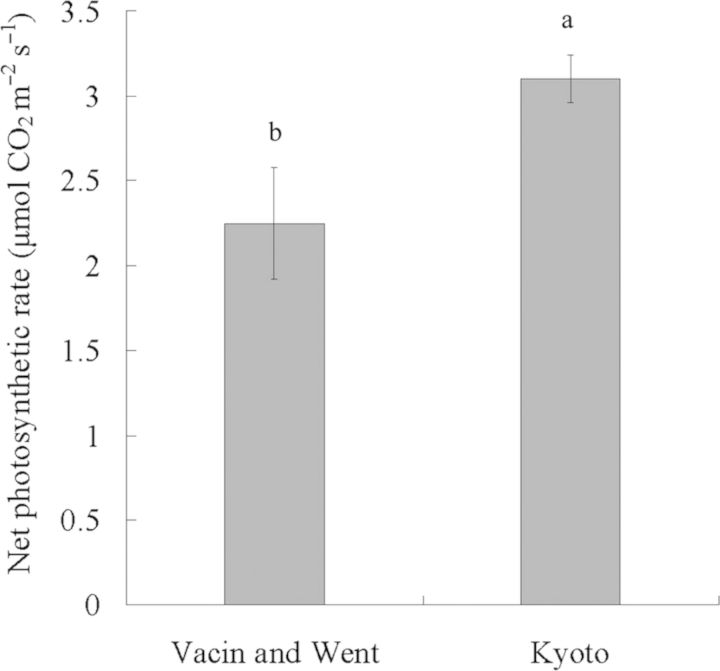
Net photosynthetic rate for single *Oncidesa* plantlet leaves grown on Vacin and Went and Kyoto media at 10 000 μmol mol^−1^ CO_2_ enrichment under high PPFD. Significant differences according to Tukey's test (*P* < 0.05) are indicated by different letters.

**Figure 5.**
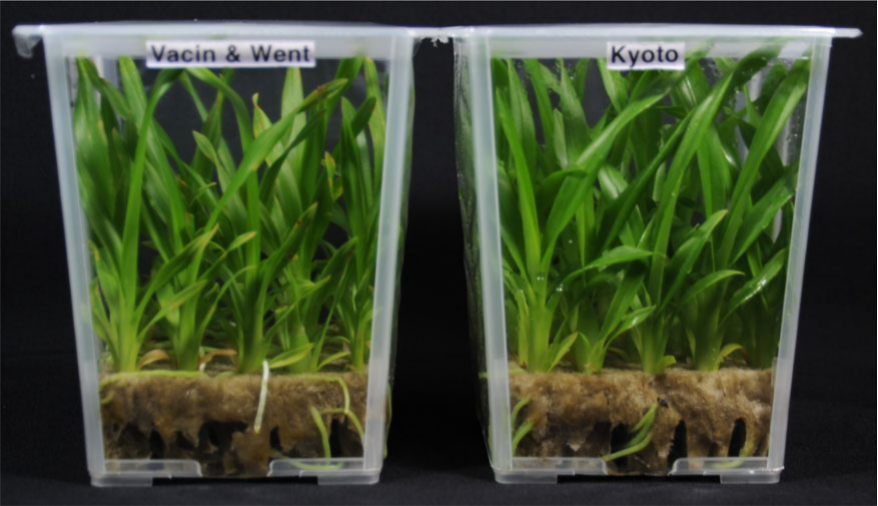
Effect of media composition on the *in vitro* growth of *Oncidesa* plantlets at 10 000 μmol mol^−1^ CO_2_ enrichment under high PPFD. Left: Vacin and Went medium; right: Kyoto medium. Significant differences according to Tukey's test (*P* < 0.05) are indicated by different letters.

It is occasionally mentioned that media developed for photomixotrophic culture are not suitable for the photoautotrophic growth and development of plants *in vitro* (Kozai *et al.* 1988; Yang *et al.* 1995). Norikane *et al.* (2010) also demonstrated that *Cymbidium* plantlets on Hyponex-based Kyoto medium, which uses compound fertilizer for plant cultivation, had a higher photosynthetic capacity at 10 000 μmol mol^−1^ CO_2_ under high PPFD than plantlets grown on modified VW medium developed for photomixotrophic culture of orchids; no symptoms such as chlorosis were observed and growth was remarkably enhanced. This was also observed in our present study. The growth of plantlets on Kyoto medium under 10 000 μmol mol^−1^ CO_2_ at high PPFD was enhanced relative to plantlets grown on VW medium; in particular, plant height, and shoot fresh and dry weights increased remarkably, although the number of leaves, root dry weight, pseudobulb formation frequency, volume, and fresh and dry weights did not differ, and the number of roots was slightly fewer (Table 2). Furthermore, the net photosynthetic rate (Fig. 4) and the SPAD value of these plantlets were higher (Table 2) and no chlorosis and browning were observed in all leaf blades (Fig. 5). Similar to a previous study on super-elevated CO_2_ (Norikane *et al.* 2010), our results also indicate that negative responses such as a decrease in photosynthetic capacity, chlorosis and browning, which were observed in plantlets grown at 10 000 μmol mol^−1^ CO_2_ under high PPFD, can be improved by altering medium components. Media composition and the nature of the carbon source strongly affect *Cymbidium* organogenic outcome (Teixeira da Silva *et al.* 2006, 2007).

**Table 2.** Effect of media composition on the *in vitro* growth of Oncidesa plantlets at 10 000 μmol mol^−1^ CO_2_ enrichment under high PPFD (60 μmol m^−2^ s^−1^). ^y^Chlorophyll content in the second leaf, counted from the top downwards, of the plantlets. ^z^Different letters within a column indicate significant differences at *P* ≤ 0.05 by Student's *t*-test. *n* = 50.

Medium	No. of leaves	No. of roots	Plant height (cm)	Pseudobulb volume (cm^3^)	Root length (cm)	Fresh weight (mg)			Dry weight (mg)			Pseudobulb formation frequency (%)	Chlorophyll content^y^ (SPAD value)
						Shoot	Pseudobulb	Root	Shoot	Pseudobulb	Root		
VW	8.3a^z^	5.8a	9.9b	1.8a	7.1a	897.1b	428.4a	360.2b	70.3b	17.1a	21.9a	10.0	32.4b
Kyoto	8.0a	5.0b	12.1a	2.9a	7.7a	1112.2a	644.7a	409.1a	78.5a	21.4a	23.2a	12.0	34.8a

## Conclusions

We have shown in this study that super-elevated CO_2_ (10 000 μmol mol^−1^) under high PPFD emitted by CCFLs enhanced the photoautotrophic growth of *Oncidesa* plantlets in the ‘Vitron’, although the upper ‘threshold limit’ would be 5000 μmol mol^−1^ CO_2_ enrichment under high PPFD before a negative impact on photosynthesis would occur. This would help to maximize the productivity and quality of *Oncidesa* plantlets cultured *in vitro*. In addition, CCFLs have several advantages over existing lighting systems used for plant tissue culture (Tanaka *et al.* 2009). In particular, CCFLs emit much less heat through their unique properties, allowing plants or cultures (culture vessels) to be placed very close to the light source, making more efficient use of the culture room and intensifying the efficiency of CO_2_ enrichment. Our results indicate that there is great hope for using super-elevated CO_2_ enrichment under CCFLs for more efficient and higher-quality commercial production of clonal orchid plantlets, which is a key objective of orchid biotechnology (Hossain *et al.* 2013; Teixeira da Silva 2013).

## Contributions by the Authors

All authors have made a substantial contribution to the manuscript and the research presented. M.T. and A.N. co-designed the experiment. M.T. and J.A.T.d.S. oversaw the experimental execution. A.N. conducted all research. A.N. and J.A.T.d.S. drafted the paper and made all edits for the revisions. All authors have seen and agreed to the submitted manuscript.

## Conflicts of Interest Statement

None declared.
